# Type I Interferon-Enhancing Effect of Cardamom Seed Extract via Intracellular Nucleic Acid Sensor Regulation

**DOI:** 10.3390/foods14152744

**Published:** 2025-08-06

**Authors:** Abdullah Al Sufian Shuvo, Masahiro Kassai, Takeshi Kawahara

**Affiliations:** 1 Division of Biological and Agricultural Sciences, Department of Science and Technology, Graduate School of Medicine, Science and Technology, Shinshu University, Minami-minowa, Kami-ina, Nagano 399-4598, Japan; 22hs552j@shinshu-u.ac.jp; 2 S&B Foods Inc., #605 Mitsui Link-Lab Shinkiba1 Shinkiba 2-3-8, Koto-ku, Tokyo 136-0082, Japan; 3 Academic Assembly, Institute of Agriculture, Shinshu University, 8304 Minami-minowa, Kami-ina, Nagano 399-4598, Japan

**Keywords:** cardamom, 1,8-cineole, type I interferon, cGAS, STING, RIG-I, TIPARP, AhR, TBK1

## Abstract

The induction of type I interferon (IFN) via intracellular nucleic acid sensors may be useful in preventing viral infections. However, little is known about the effect of natural plant materials on sensor responses. We previously found that cardamom (*Elettaria cardamomum* (L.) Maton) seed extract (CSWE) enhanced type I IFN expression and prevented influenza virus infection. In this study, we investigated the effect of CSWE on type I IFN responses using intracellular nucleic acid sensor molecules. Human lung epithelial A549 cells were treated with CSWE and transfected with poly(dA:dT) or poly(I:C) using lipofection. CSWE and 1,8-cineole, the major CSWE components, dose-dependently induced type I IFNs and IFN-stimulated genes in both poly(dA:dT)- and poly(I:C)-transfected A549 cells. The type I IFN-enhancing effect of CSWE was dependent on the stimulator of interferon genes (STING), whereas the effect of 1,8-cineole was independent of STING and mediated by the down-regulation of 2,3,7,8-tetrachlorodibenzo-*p*-dioxin (TCDD)-inducible poly-ADP-ribose polymerase expression. Our study suggests that CSWE has the potential to act as a beneficial antiviral agent by enhancing homeostatic type I IFN production.

## 1. Introduction

Pattern recognition receptors (PRRs) recognize molecular patterns the body identifies as threats, such as damage-associated molecular patterns (DAMPs) and pathogen-associated molecular patterns (PAMPs), and are responsible for the innate immune response. PRRs are present on the cell surface (including the inner surface of endosomes) and in the cytoplasm, where they play a role in the recognition of infectious pathogens [1,2]. Several PRRs activate signaling pathways that adapt to viral infections. Once a viral infection has been established, intracellular nucleic acid sensor molecules, including cyclic GMP-AMP synthase (cGAS) and retinoic acid-inducible gene-I (RIG-I), recognize virus-derived double-stranded DNA (dsDNA) and double-stranded RNA (dsRNA), respectively, in the cytoplasm [3,4,5]. These sensor molecules are believed to provide defensive benefits by inducing the production of various cytokines against different viruses and at different stages of infection based on their distinct nucleic acid structures.

One of the most important cytokines involved in viral infections is type I interferon (IFN). Shortly after viral infection, type I IFNs are released to clear the viral particles by inhibiting their replication. Type I IFNs play an important antiviral role by inhibiting viral replication in infected cells, activating and enhancing antigens, and triggering adaptive immune responses through direct and indirect action on B and T cells [6]. Furthermore, type I IFN secretion enhances the production of many IFN-stimulated genes (ISGs) with antiviral properties. ISGs are involved in processes such as protein degradation, post-translational regulation of gene expression, activation and repression of transcription, modulation of immune cells, release of cytokines, and apoptosis. Among other type I IFNs, IFN-β has an immune-modulatory effect on respiratory viral infections and activates the immune response in infected lung tissue [7,8,9].

Traditional and modern medication systems rely on plant-based remedies to treat a wide range of diseases, including viral respiratory infections. Medicinal plants and herbs, along with their bioactive components, are known as green medicines and have been traditionally used for a long time. Plant-based bioactive components are a rich source of synthetic antiviral agents that attract pharmaceutical companies to explore new compounds from nature in an environmentally friendly manner [10]. Several plant materials and specific bioactive components are used in antiviral products. Moreover, many of these materials interfere with the different stages of the viral life cycle in various ways, including the induction of IFN to modulate immunity [11,12]. However, little is known about the functional properties of plant materials in terms of their effects on type I IFN induction mediated by intracellular nucleic acid sensors. Recently, we found that cardamom (*Elettaria cardamomum* (L.) Maton) seed extract induced type I IFNs and IFN-stimulated antiviral proteins, thereby inhibiting human influenza A virus infection (unpublished data). There is growing evidence that the activation of both the cGAS-stimulator of interferon genes (STING) and RIG-I-mitochondrial antiviral signaling protein (MAVS) pathways is necessary for the suppression of influenza A virus [13,14,15].

In this study, we investigated the effect of cardamom hot-water extract (CSWE) on type I IFN responses induced by intracellular nucleic acid sensors using poly(I:C) and poly(dA:dT) in human lung epithelial cells.

## 2. Materials and Methods

### 2.1. Preparation of CSWE

The cardamom seeds were provided by S&B Foods Inc. in Tokyo, Japan. Detailed information of the plant material with collection data has been deposited in S&B Foods Inc. (Tokyo, Japan, Lot No. 20170831). Cardamom seed powder (10 g) was dissolved in 10 mL of water to make an aqueous solution. Next, the extraction was performed by heating to 100 °C for 1 h while constantly swirling it with a stirrer. The resultant extract was then centrifuged and the supernatant freeze-dried to obtain CSWE powder. The CSWE powder was then dissolved in water for use in subsequent experiments.

### 2.2. Cells and Cell Culture

The human lung adenocarcinoma cell line (A549) was obtained from the JCRB Cell Bank (Osaka, Japan). The cells were cultured in Eagle’s minimal essential medium (EMEM; FUJIFILM Wako Pure Chemical, Osaka, Japan) supplemented with 10% (*v*/*v*) fetal bovine serum (FBS; Cytiva, Tokyo, Japan), 100 U/mL penicillin, and 100 μg/mL streptomycin solution (FUJIFILM Wako Pure Chemical). The A549-Dual cells were purchased from InvivoGen (San Diego, CA, USA) and cultured in Dulbecco’s modified Eagle medium (DMEM; FUJIFILM Wako Pure Chemical) supplemented with 10 µg/mL blasticidin (InvivoGen) and 100 g/mL Zeocin (InvivoGen). These cells were maintained in a humidified environment of 5% CO_2_ and 95% air at 37 °C.

### 2.3. Transfection with Poly(dA:dT) and Poly(I:C)

A549 cells were seeded at 1 × 10^5^ cells/mL/well in a 24-well cell culture plate and incubated for 24 h. The cells were treated with CSWE at concentrations at 0–100 µg/mL or 1,8-cineole at 0–1000 nM. The cells were then stimulated with the mixture containing 0.1 µg/mL of either poly(dA:dT) (InvivoGen) or poly(I:C) LMW (InvivoGen), as well as Lipofectamine 2000 (Thermo Fisher Scientific, Waltham, MA, USA) in Opti-MEM (Thermo Fisher Scientific) for 4 h. The cells were then cultured in EMEM containing 10% FBS under a humidified environment of 5% CO_2_ and 95% air at 37 °C for 6 h for reverse transcription and quantitative polymerase chain reaction (PCR) analysis, or for 48 h for enzyme-linked immunosorbent assay (ELISA).

### 2.4. Reverse Transcription and Quantitative PCR

Total RNA was isolated from cell lysates using TRI Reagent (Merck) according to the manufacturer’s instructions. The obtained RNA was then reverse transcribed at 42 °C for 50 min using M-MLV reverse transcriptase (Thermo Fisher Scientific). A random primer pd (N)_9_ (10 pmol/μL, Takara Bio, Shiga, Japan) was used in a PTC-200 thermal cycler (MJ Research, Waltham, MA, USA). The obtained cDNA was analyzed by quantitative real-time PCR using THUNDERBIRD Next SYBR qPCR Mix (Toyobo, Osaka, Japan). The reaction consisted of a preheating cycle (95 °C, 10 min), followed by 40 cycles of denaturation (95 °C, 10 s) and primer annealing/extension (55 °C, 30 s) on the Eco Illumina Real-Time PCR System (Illumina, San Diego, CA, USA). The nucleotide sequences of the forward and reverse primers are shown in Table 1. The results were analyzed using the ΔΔCt method with Eco Real-Time PCR System software version 4.0 (Illumina). The amount of PCR product was normalized to the expression level of glyceraldehyde-3-phosphate dehydrogenase (GAPDH).

### 2.5. ELISA

The culture supernatant of A549 cells after the above treatment was collected and centrifuged at 10,000 rpm for 10 min. The supernatant was then filtered using a 0.20 µm-pore size nitrocellulose membrane filter. The concentration of IFN-β in the culture supernatant was detected using a Human IFN-β ELISA kit (Abcam, Cambridge, UK) according to the manufacturer’s instructions.

### 2.6. Gas Chromatography–Mass Spectrometry (GC-MS) Analysis

The constituents of CSWE were analyzed using GC-MS. To prepare the sample, 0.5 g of CSWE was placed in a centrifuge tube and 2 mL of water was added. The mixture was allowed to swell for 20 min. Then, 25 mL of acetone was added and extracted with shaking for 5 min. Then, 1 mL of the supernatant (equivalent to 0.1 g of the sample) was diluted with 50 mL of hexane/acetone (1:1) and filtered.

GC was performed using a GCMS-8040 instrument (Shimadzu, Kyoto, Japan) equipped with a Stabilwax GC Capillary Column (60 m, 0.25 mm i.d., 0.15 μm film thickness; Restek, Bellefonte, PA, USA). The injected volume was 1 μL using the splitless method. The vaporization chamber temperature was set to 230 °C. The column temperature protocol was started at 40 °C (held for 1 min), ramped at 5 °C/min to 100 °C, then ramped at 2 °C/min to 130 °C, and finally ramped at 10 °C/min to 230 °C and held for 10 min. Helium was used as the carrier gas at a flow rate of 25.8 cm/s with a constant linear velocity.

MS analyses were performed in electron impact (EI) mode with an ionization energy of 70 eV. The mass spectra were recorded between a range of 80 and 180 atomic mass units. The identification of 1,8-cineole was based on its retention index using an analytical standard with 99.0% purity (Sigma-Aldrich Merck KGaA, Darmstadt, Germany), and other components were identified by matching their mass spectral peaks with a mass spectrum library.

### 2.7. Liquid Chromatography–Mass Spectrometry (LC-MS/MS) Analysis

The amount of 1,8-cineole was estimated using LC-MS/MS analysis. For sample preparation, 0.1 g of the sample was placed in a measuring flask, 0.5 mL of water was added, and the mixture was allowed to swell for 20 min. Then, 4.5 mL of acetone was added to the extracted sample, it was sonicated for 15 min, and then it was filtered.

LC-MS analysis was performed using an LCMS8060 instrument (Shimadzu) equipped with a ZORBAX Eclipse Plus C18 column (No. 49, 100 mm length, 2.1 mm i.d., 1.8 µm particle size; Agilent Technologies, Santa Clara, CA, USA) and 1 μL of the sample was injected. The mobile phase was constructed of (A) 0.1% formic acid solution and (B) acetonitrile, and separation was performed using the following program: 0–0.5 min, 70% B, varied linearly to 95% B for 5 min, and followed by a 3 min post-run. The flow rate was 0.3 mL/min. The column temperature was set to 40 °C. Retention time was 2.6 min. The monitoring ions were 137.1 > 81.1 (10 eV) and 95.1 (10 eV).

### 2.8. Reporter Assay

A549-Dual cells were seeded at 5 × 10^4^ cells/100 μL/well in a white 96-well cell culture plate (Corning, Corning, NY, USA) for 24 h. Then, a different concentration of CSWE was added to the culture media for another 24 h followed by the stimulation with 0.1 µg/mL poly(dA:dT) or 0.1 µg/mL poly(I:C) LMW in Opti-MEM for 4 h. The cells were then allowed to culture for another 8 h to evaluate IRF reporter activity or for 24 h to evaluate NF-κB reporter activity. To evaluate IRF reporter activity, 20 µL of QUANTI-Luc 4 Lucia/Gaussia (InvivoGen) was added to the cell culture and chemiluminescence was measured using a SpectraMax L (Molecular devices, San Jose, CA, USA). To evaluate NF-κB reporter activity, culture supernatant was added to the QUANTI-Blue solution (InvivoGen) for 30 min and measured the absorbance at 655 nm using an iMark microplate reader (Bio-Rad, Hercules, CA, USA).

### 2.9. Inhibition Experiment

A549 cells were seeded at 2 × 10^5^ cells/mL/well in a 24-well cell culture plate and incubated for 24 h. Then, the cells were treated with 20 µM of H-151 (MedChemExpress, Monmouth Junction, NJ, USA) dissolved in DMSO for 4 h. Next, the cells were then treated with CSWE or 1,8-cineole for 4 h. The cells were stimulated with poly(dA:dT) for 4 h and collected for quantitative PCR analysis.

### 2.10. Statistical Analysis

The data were statistically analyzed using one-way analysis of variance (ANOVA) and the Tukey–Kramer multiple comparison test. The analysis was performed using Excel 2019 (Microsoft, Redmond, WA, USA) with the Statcel4 add-in software from OMS Publishing (Tokyo, Japan). Results with a *p*-value of less than 0.05 were considered statistically significant.

## 3. Results

### 3.1. CSWE Enhanced Induction of Type I IFNs and ISGs in A549 Cells Transfected with Poly(dA:dT)

**Figure 1:**
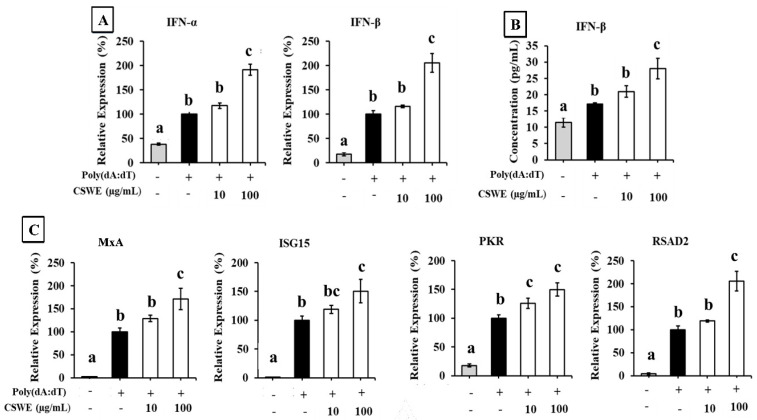
The effect of CSWE on type I IFN and ISG expressions in A549 cells transfected with poly(dA:dT). (**A**) IFN-α and -β mRNA expression in A549 cells transfected with poly(dA:dT). (**B**) IFN-β production in culture supernatant of A549 cells transfected with poly(dA:dT). (**C**) ISG (MxA, ISG15, PKR, and RSAD2) expressions in A549 cells transfected with poly(dA:dT). Data were represented as the mean ± standard deviation (SD). Different letters denote significant differences at *p* < 0.05 (n = 3).

Figure 1 shows the effect of CSWE on the expression of type I IFNs in A549 cells transfected with poly(dA:dT). Transfection with poly(dA:dT) significantly increased the expression of IFN-α and IFN-β in A549 cells. CSWE enhanced the expression of both type I IFNs induced by poly(dA:dT) in a dose-dependent manner (Figure 1A). Treatment with CSWE also increased the concentration of IFN-β in the culture supernatant. (Figure 1B). The expression of ISGs induced by type I IFNs (MxA, ISG15, PKR, and RSAD2) was also enhanced by CSWE treatment (Figure 1C).

### 3.2. CSWE Enhanced Induction of Type I IFNs and ISGs in A549 Cells Transfected with Poly(I:C)

**Figure 2:**
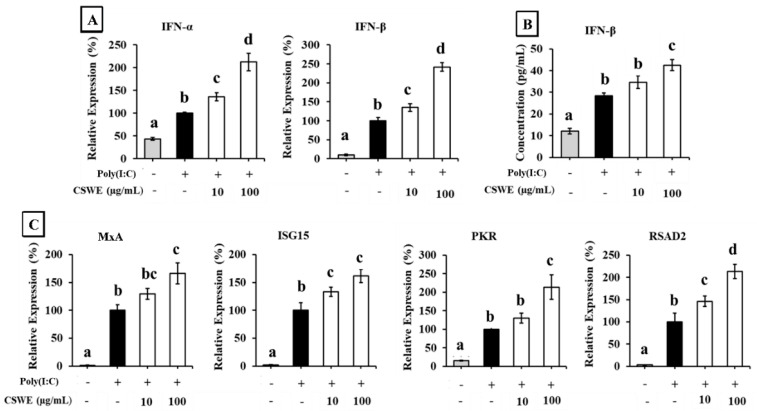
The effect of CSWE on type I IFN and ISG expressions in A549 cells transfected with poly(I:C). (**A**) IFN-α and -β mRNA expression in A549 cells transfected with poly(I:C). (**B**) IFN-β production in culture supernatant of A549 cells transfected with poly(I:C). (**C**) ISG (MxA, ISG15, PKR, and RSAD2) expressions in A549 cells transfected with poly(I:C). Data were presented as the mean ± SD. Different letters denote significant differences at *p* < 0.05 (n = 3).

The effect of CSWE on the type I IFN expression in A549 cells transfected with poly(I:C) is shown in Figure 2. Transfection with poly(I:C) significantly increased the expression of IFN-α and IFN-β in A549 cells. CSWE enhanced the expression of both type I IFNs induced by poly(I:C) in a dose-dependent manner (Figure 2A). Treatment with CSWE also increased the concentration of IFN-β protein in the culture supernatant (Figure 2B). CSWE also enhanced the expression of ISGs (Figure 2C).

### 3.3. CSWE Enhanced Induction of Type I IFNs and ISGs in A549 Cells

**Figure 3:**
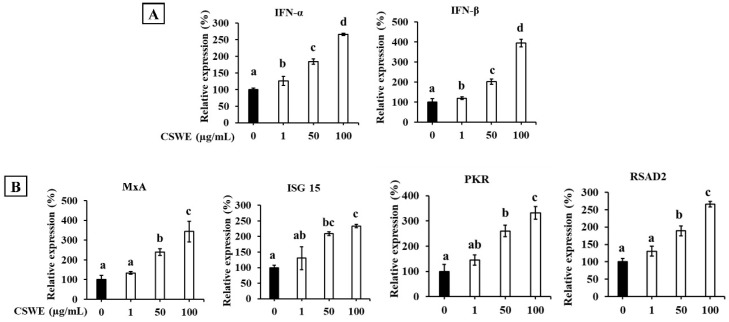
The effect of CSWE on type I IFN and ISG expression in A549 cells. (**A**) IFN-α and -β mRNA expression in A549 cells. (**B**) ISG (MxA, ISG15, PKR, and RSAD2) mRNA expression in A549 cells. Data were presented as the mean ± SD. Different letters denote significant differences at *p* < 0.05 (n = 3).

The effect of CSWE on type I IFN expression in A549 cells is shown in Figure 3. CSWE enhanced the expression of type I IFNs in a dose-dependent manner (Figure 3A). CSWE treatment also enhanced the expression of ISGs (Figure 3B).

### 3.4. GC-MS Analysis of Plant-Based Substance

GC/MS analysis of CSWE provided the chromatogram shown in Supplementary Figure S1. Two major compounds, 1,8-cineole (peak 1) and α-terpinyl acetate (peak 5), were detected, as previously reported [16,17]. In addition, we identified acetic acid (peak 3), terpinen-4-ol (peak 4), and geraniol (peak 6). The concentration of 1,8-cineole was estimated to be 25.3 nM by LC-MS/MS analysis.

### 3.5. 1,8-Cineole Enhanced Expression of Type I IFNs and ISGs in A549 Cells Transfected with Poly(dA:dT)

**Figure 4:**
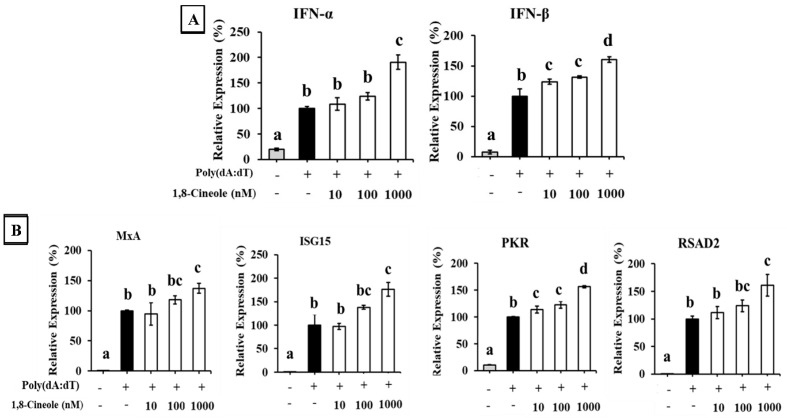
The effect of 1,8-cineole on type I IFN and ISG expression in A549 cells transfected with poly(dA:dT). (**A**) IFN-α and -β mRNA expression in A549 cells transfected with poly(dA:dT). (**B**) ISG (MxA, ISG15, PKR, and RSAD2) mRNA expression in A549 cells transfected with poly(dA:dT). Data were presented as the mean ± SD. Different letters denote significant differences at *p* < 0.05 (n = 3).

The effect of 1,8-cineole on the expression of type I IFNs in A549 cells transfected with poly(dA:dT) is shown in Figure 4. Transfection with poly(dA:dT) significantly induced the expression of IFN-α and IFN-β in A549 cells. Additionally, 1,8-cineole enhanced the expression of both poly(dA:dT)-induced type I IFNs in a dose-dependent manner (Figure 4A). The expression of ISGs was also enhanced by 1,8-cineole treatment (Figure 4B).

### 3.6. 1,8-Cineole Enhanced Expression of Type I IFNs and ISGs in A549 Cells Transfected with Poly(I:C)

**Figure 5:**
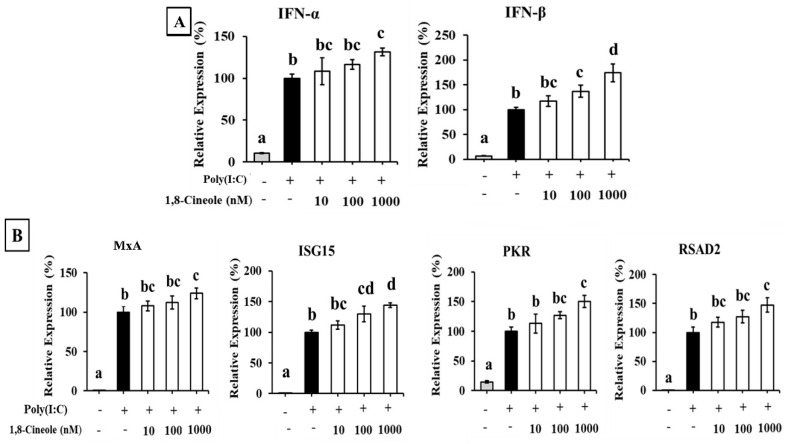
The effect of 1,8-cineole on type I IFN and ISG expression in A549 cells transfected with poly(I:C). (**A**) IFN-α and -β mRNA expression in A549 cells transfected with poly(I:C). (**B**) ISG (MxA, ISG15, PKR, and RSAD2) mRNA expression in A549 cells transfected with poly(I:C). Data were presented as the mean ± SD. Different letters denote significant differences at *p* < 0.05 (n = 3).

The effect of 1,8-cineole on type I IFN expression in A549 cells transfected with poly(I:C) is shown in Figure 5. Transfection with poly(I:C) significantly induced the expression of IFN-α and IFN-β in A549 cells. Additionally, 1,8-cineole enhanced poly(I:C)-induced type I IFN expression in a dose-dependent manner (Figure 5A). The expression of ISGs was also enhanced by 1,8-cineole treatment (Figure 5B).

### 3.7. 1,8-Cineole Enhanced Expression of Type I IFN and ISGs in A549 Cells

**Figure 6:**
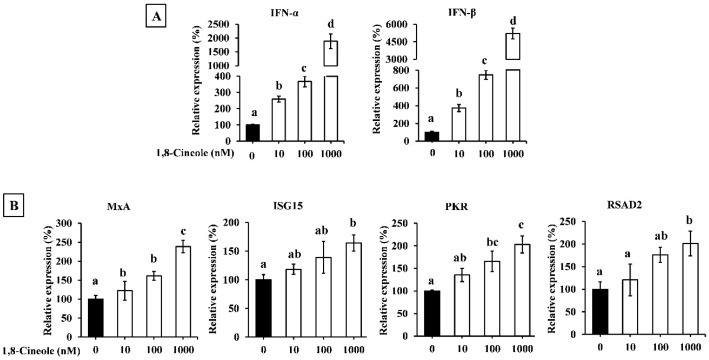
The effect of 1,8-cineole on type I IFN expression in A549 cells. IFN-α and -β (**A**) as well as ISGs (**B**) mRNA expression in A549 cell treated with 1,8-cineole. Data were presented as the mean ± SD. Different letters denote significant differences at *p* < 0.05 (n = 3).

Figure 6 shows the effect of 1,8-cineole on the expression of type I IFNs in A549 cells. As shown, 1,8-cineole enhanced the expression of type I IFNs in a dose-dependent manner (Figure 6A). The expression of ISGs was also enhanced by 1,8-cineole treatment (Figure 6B).

### 3.8. CSWE and 1,8-Cineole Significantly Enhanced IRF but Not NF-κB Reporter Activities in A549-Dual Cells

**Figure 7:**
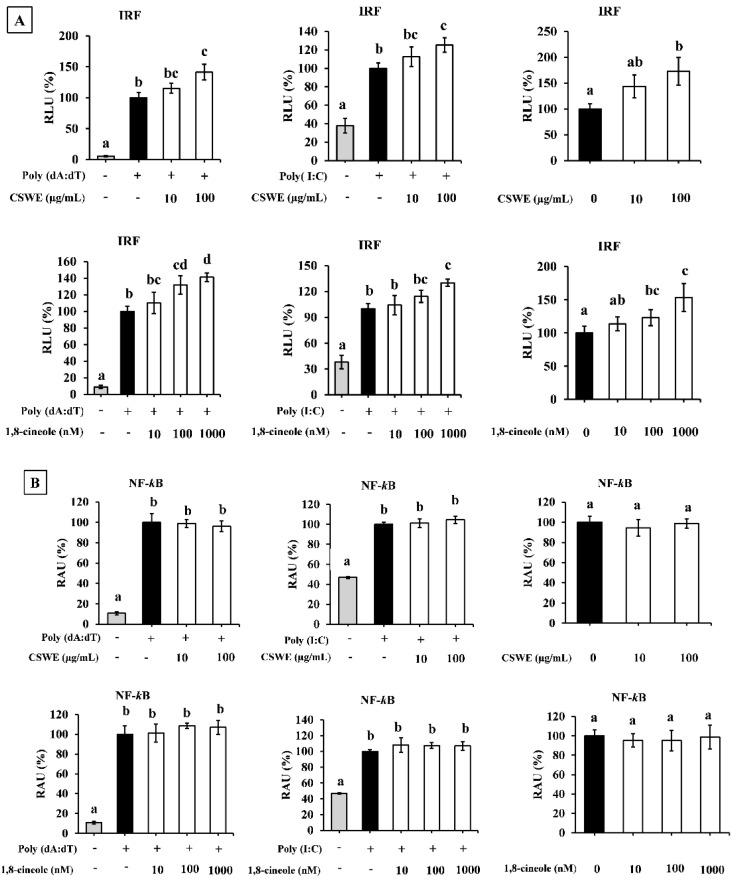
The effect of CSWE and 1,8-cineole on IRF and NF-κB reporter activity in A549-Dual cells transfected with poly(dA:dT) and poly(I:C). (**A**) IRF reporter activity in A549-Dual cells. RLU = Relative luminescence unit. Different letters denote significant differences at *p* < 0.05 (n = 3). (**B**) NF-κB reporter activity in A549-Dual cells. RAU = relative absorbance unit. Different letters denote significant differences at *p* < 0.05 (n = 3).

Transfection with poly(dA:dT)- and poly(I:C)-induced IRF and NF-κB reporter activities in A549-Dual cells compared to the unstimulated condition. Both CSWE and 1,8-cineole significantly enhanced IRF-mediated reporter activity in unstimulated cells and the cells transfected with poly(dA:dT) and poly(I:C) (Figure 7A). On the other hand, CSWE and 1,8-cineole had no significant effect on NF-κB-mediated reporter activity in A549-Dual cells (Figure 7B).

### 3.9. Type I IFN Induction in A549 Cells by CSWE but Not by 1,8-Cineole Was Inhibited by STING Inhibitor

**Figure 8:**
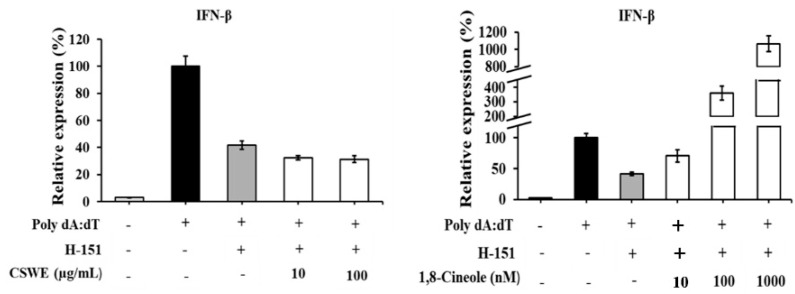
The effect of STING inhibitor on type I IFN-enhancing effect of CSWE and 1,8-cineole in A549 cells transfected with poly(dA:dT). IFN-β mRNA expression in A549 cells transfected with poly(dA:dT). Data were presented as the mean ± SD. Different letters denote significant differences at *p* < 0.05 (n = 3).

Figure 8 shows the effect of the STING inhibitor H-151 on CSWE- and 1,8-cineole-induced IFN-β in A549 cells. CSWE-induced IFN-β expression was significantly inhibited in the presence of H-151. The 1,8-cineole-induced IFN-β expression was not affected by H-151.

### 3.10. CSWE and 1,8-Cineole-Suppressed TIPARP Expression

**Figure 9:**
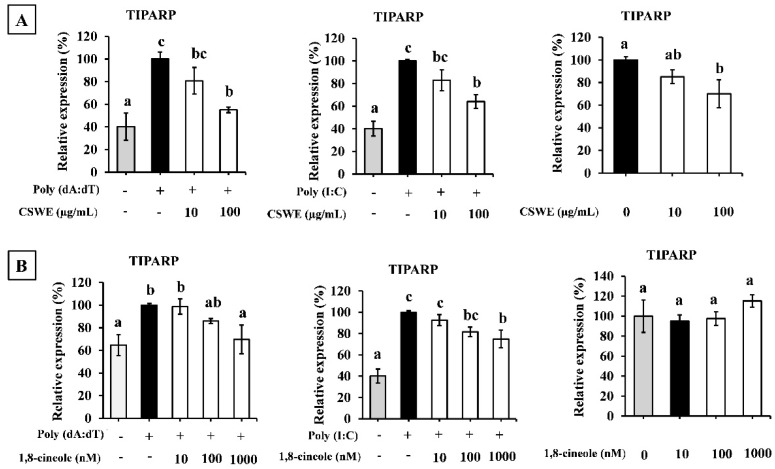
The effect of CSWE and 1,8-cineole on TIPARP expression in A549 cells. (**A**) The mRNA expression of TIPARP in A549 cell pre-treated with CSWE as well as transfected with poly(dA:dT), poly(I:C), and unstimulated condition. (**B**) TIPARP mRNA expression in A549 cell pre-treated with 1,8-cineole as well as transfected with poly(dA:dT), poly(I:C), and unstimulated condition. Data were presented as the mean ± SD. Different letters denote significant differences at *p* < 0.05 (n = 3).

To observe the effects of CSWE and 1,8-cineole on TIPARP expression, A549 cells were transfected with poly(dA:dT) and poly(I:C) where the expression was compared with an unstimulated state. Figure 9A shows that CSWE reduces the expression of TIPARP on both poly(dA:dT) and poly(I:C) transfected conditions, while 1,8-cineole also suppresses the expression of TIPARP on both poly(dA:dT) and poly(I:C) transfected conditions (Figure 9B) in A549 cells.

## 4. Discussion

In this study, we investigated the effects of CSWE on intracellular nucleic acid sensor-mediated pathways related to IFN induction using human lung epithelial A549 cells, a target of human influenza A virus infection, which exhibit high levels of IFN expression upon viral infection [18]. We also used commercially available double-stranded (ds)DNA and dsRNA. As shown in Supplementary Figure S1, 1,8-cineole and α-terpinyl acetate, which are reported as major components in the water extracted from cardamom seeds under various conditions [19,20], are detected as the components of CSWE. Monoterpene cyclic ether, 1,8-cineole, known as eucalyptol, has been focused as an anti-inflammatory adjuvant for antiviral vaccines and a modulator of cytokine responses induced by PRRs on the surface of cell membranes and endosomes [11,12,13,14,15,16,17,18,19,20,21,22,23]. Thus, we focused on the effect of CSWE and the involvement of 1,8-cineole on the function of CSWE in this study. Of course, we do not intend to exclude the possibility that other components, including α-terpinyl acetate, may exhibit similar effects. Further study is needed to evaluate the possibility of the involvement of other detected compounds in the revealed function of CSWE in this study.

First, we used the viral dsDNA analog poly(dA:dT) to evaluate the effect of CSWE on the production of type I IFNs via intracellular DNA sensors. Transfection with dsDNA or dsRNA induces the release of type I IFNs through intracellular nucleic acid sensors by mimicking a pseudo-viral entry state, as previously described [24]. A549 cells pretreated with CSWE and stimulated by transfection with poly(dA:dT) exhibited a dose-dependent induction of type I IFN mRNA and IFN-β protein expression compared to control cells stimulated with poly(dA:dT) alone (Figure 1A,B). The expression levels of representative ISGs (MxA, ISG15, PKR, and RSAD2) were also increased by CSWE treatment in poly(dA:dT)-transfected A549 cells (Figure 1C). We further investigated the effect of CSWE on type I IFN via intracellular RNA sensors using poly(I:C), a viral dsRNA analog, in the same manner. CSWE also enhanced the expression of type I IFNs (Figure 2A), IFN-β protein production (Figure 2B), and ISG expression (Figure 2C) in a dose-dependent manner.

Poly(dA:dT) is a synthetic analog of B-form double-stranded DNA that activates the cGAS-STING signaling pathway to induce type I IFNs [25,26]. In contrast, poly(I:C) is a synthetic dsRNA consisting of a polymer strand of inosinic and cytidylic acids that activates the RIG-I- or melanoma differentiation-associated gene 5 (MDA5)-MAVS signaling pathway [27,28,29]. RIG-I and MDA5 recognize dsRNA depending on the length of the molecule, and poly(I:C) LMW, with an average size of 0.2–1 kb, used in the present study, is primarily recognized by RIG-I [28]. Our results suggested that CSWE enhanced type I IFN responses induced by two different signaling pathways: cGAS-STING and RIG-I-MAVS. However, because poly(dA:dT) is transcribed into dsRNA containing a 5′-triphosphate group (5′-ppp-dsRNA) with the help of RNA polymerase III and activates the RIG-I pathway [24], it is debatable whether CSWE activates the two signaling pathways independently. Additionally, CSWE alone may induce type I IFNs regardless of the activation status of A549 cells. Thus, the ability of CSWE to induce type I IFNs was evaluated in unstimulated A549 cells. CSWE induced type I IFNs and ISGs without stimulation by poly(dA:dT) or poly(I:C) (Figure 3).

Therefore, we investigated the effect of 1,8-cineole on the type I IFN response in cells stimulated with poly(dA:dT) and poly(I:C). A549 cells pretreated with 1,8-cineole exhibited increased IFN-α and IFN-β mRNA expression and ISG protein expression (Figure 4 and Figure 5). Furthermore, 1,8-cineole induced type I IFN production, similar to CSWE, even in unstimulated A549 cells (Figure 6). These results suggest that 1,8-cineole is an active component of CSWE that promotes the expression of type I IFNs and ISG proteins.

However, the mechanism of action that induces type I IFN and ISG proteins remains unclear. IFN regulatory factor (IRF)3 and nuclear factor-kappa B (NF-κB) are transcription factors that induce type I IFNs. To monitor the effect of CSWE and 1,8-cineole on IRF3 and NF-κB, we used A549-Dual cells, a subclone of the A549 cell line via stable integration of IRF3- and NF-κB-inducible reporter constructs. Stimulation with poly(dA:dT) and poly(I:C) increased both IRF3 and NF-κB reporter activity. In this condition, CSWE enhanced IRF reporter activity but not NF-κB reporter activity (Figure 7). These results suggest that CSWE enhances the type I IFN response induced by the activation of the IRF pathway. Furthermore, we evaluated the effects of CSWE and 1,8-cineole in A549 cells pretreated with H-151, a selective and covalent STING antagonist [30]. The CSWE’s IFN-β-enhancing effect was inhibited by H-151, whereas that of 1,8-cineole was unaffected (Figure 8). These results suggest that CSWE contains active component(s) in addition to 1,8-cineole, and that its type I IFN-inducing activity was dependent on the STING pathway. Furthermore, it indicates that type I IFN induction by 1,8-cineole alone is independent of the STING pathway.

Lee et al. [31] reported that 1,8-cineole targeted aryl hydrocarbon receptors (AhRs), and prevented UVB-induced skin carcinogenesis. Furthermore, Taisho et al. [32] reported that AhR signaling suppressed type I interferon-mediated antiviral response by inducing 2,3,7,8-tetrachlorodibenzo-p-dioxin (TCDD)-inducible poly-ADP-ribose polymerase (TIPARP). Thus, we hypothesized that 1,8-cineole suppresses TIPARP expression via AhR and evaluated its effects. As shown in Figure 9, stimulation of TIPARP expression by poly(dA:dT) and poly(I:C) was significantly reduced in A549 cells treated with CSWE and 1,8-cineole. Additionally, CH-223191, an AhR inhibitor, increased type I IFNs in A549 cells similarly to 1,8-cineole (Supplementary Figure S2). These results suggest that AhR inhibition is involved in the type I IFN-inductive effect of 1,8-cineole. TIPARP is a mono-ADP-ribosyltransferase enzyme that is induced via the xenobiotic response element (XRE) target sequence by AhR activation. It modifies AhR activation by catalyzing the transfer of a single ADP-ribose unit from NAD^+^ to AhR, thereby functioning as a feedback molecule in AhR signaling [33]. TIPARP also interacted with TBK1 and suppressed its activity by ADP-ribosylation. [32]. TBK1 is an essential component of IRF3-mediated signaling [34] and induces innate immunity through both the cGAS-STING and RIG-I-MAVS signaling pathways [25,26]. AhR activation suppresses the constant defensive function against viral infection by suppressing TBK1. Therefore, our results suggested that CSWE released the inhibitory state of TBK1 induced by 1,8-cineole and efficiently induced type I IFNs by activating the STING pathway via another active component. The reason why type I IFN expression was induced even with 1,8-cineole alone remains unclear. A recent study suggests that type I IFNs are secreted constitutively at low concentrations despite viral infection [35]. Although the mechanisms underlying its generation remain unclear, such pre-priming type I IFN production has been shown to be important in enhancing subsequent antiviral responses [36]. The induction of IFN by 1,8-cineole alone may be due to the activation of such endogenous type I IFN-inducing signaling.

In conclusion, we found that CSWE and its component, 1,8-cineole, enhanced type I IFN responses, including those induced by poly(dA:dT) and poly(I:C). To the best of our knowledge, this is the first report demonstrating that plant food material and their active components enhance the induction of type I IFN through both cGAS-MAVS-STING and RIG-I-MAVS signaling pathways, which are intracellular nucleic acid sensors. These defense mechanisms, mediated by intracellular nucleic acid sensors, are expected to inhibit not only influenza viruses, but also a wide range of viral infections [37]. Our study suggests that CSWE has the potential to act as a beneficial antiviral agent by enhancing the production of endogenous and exogenous type I IFN production.

## Figures and Tables

**Table 1:** Primer sequences used in study.

Target Protein	Sequence (5′-3′)		Size (bp)
GAPDH	Forward	AACGGATTTGGTCGTATTGG	90
	Reverse	AATGAAGGGGTCATTGATGG	
IFN-α1	Forward	CCTCGCCCTTTGCTTTACTG	60
	Reverse	GAGAGCAGCTTGACTTGCAG	
IFN-β	Forward	CAACCTTTCGAAGCCTTTGC	50
	Reverse	CAACCTTTCGAAGCCTTTGC	
MxA	Forward	TTCATGCTCCAGACGTACGG	188
	Reverse	TGTGGTTAACCGGGGAACTG	
ISG15	Forward	CTCTGAGCATCCTGGTGAGGAA	136
	Reverse	AAGGTCAGCCAGAACAGGTCGT	
PKR	Forward	GAAGTGGACCTCTACGCTTTGG	106
	Reverse	TGATGCCATCCCGTAGGTCTGT	
RSAD2	Forward	CCAGTGCAACTACAAATGCGGC	153
	Reverse	CGGTCTTGAAGAAATGGCTCTCC	
TIPARP	Forward	GATTCTCAGGAGCACTTGGAAAG	153
	Reverse	TGGTGTGGACAGCCTTCGTAGT	

## Data Availability

The original contributions presented in this study are included in the article/Supplementary Materials. Further inquiries can be directed to the corresponding author.

## Supplementary Materials

The following supporting information can be downloaded at: https://www.mdpi.com/article/10.3390/foods14152744/s1, Figure S1: Gas chromatogram profile of CSWE. The numbers shown in chromatogram represent 1 for 1,8-cineole, 2 for (internal standard), 3 for acetic acid, 4 for terpine-4-ol, 5 for α-terpinyl acetate, and 6 for geraniol. Figure S2. The effect of CH223191 on TIPARP expression in A549 cells. TIPARP expression in A549 cells treated with CH-223191 for 6 hours. Data were presented as the mean ± SD. Different letters denote significant differences at *p* < 0.05 (n = 3).
