# Supplementary material for: Type I Interferon-Enhancing Effect of Cardamom Seed Extract via Intracellular Nucleic Acid Sensor Regulation
Source: Foods. 2025 Aug 6;14(15):2744. doi: 10.3390/foods14152744 (PMC12346419; doi:10.3390/foods14152744)
Supplement: Supplementary file 1 [file foods-14-02744-s001.zip › foods-3714823-supplementary.pdf]

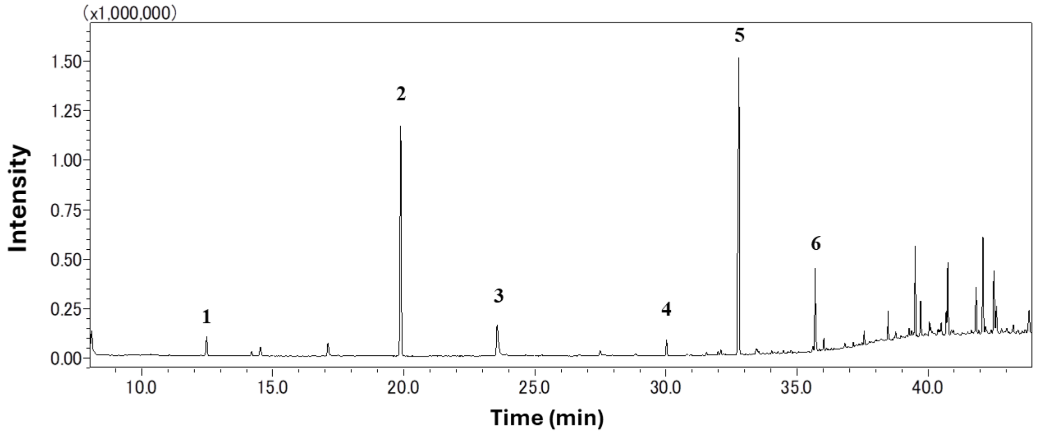

Supplementary Figure S1. Gas chromatogram profile of CSWE

The numbers shown in chromatogram represent 1 for 1,8-cineole, 2 for (internal standard), 3 for acetic acid, 4 for terpine-4-ol, 5 for α-terpinyl acetate, and 6 for geraniol.

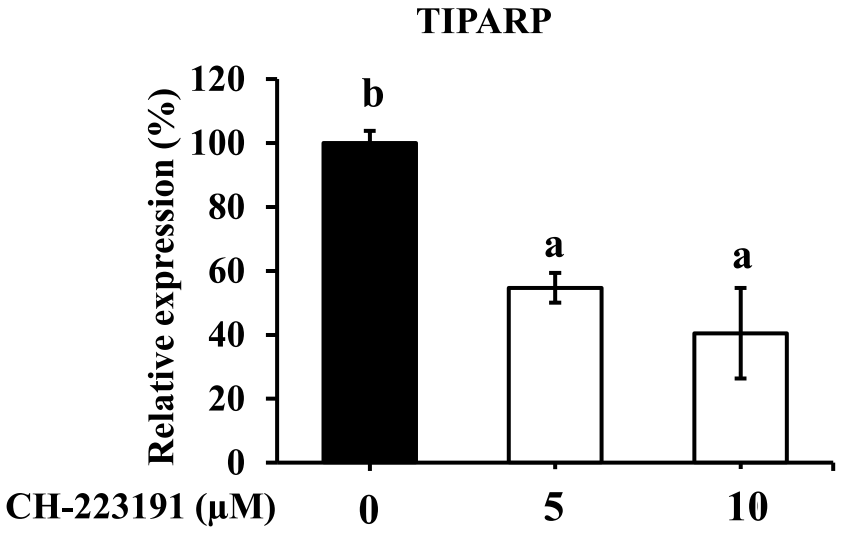

Supplementary Figure S2. The effect of CH223191 on TIPARP expression in A549 cells

TIPARP expression in A549 cells treated with CH-223191 for 6 hours. Data were presented as the mean ± SD. Different letters denote significant differences at $p < 0.05$ (n = 3).
